# Clinical and Therapeutic Characteristics of Pituitary TSH-Secreting Adenoma in Adolescent-Onset Patients: Six Case Studies and Literature Review

**DOI:** 10.3389/fendo.2021.771673

**Published:** 2021-12-23

**Authors:** Yamei Yang, Jie Liu, Kan Deng, Lin Lu, Huijuan Zhu, Xiaolan Lian, Xinjie Bao, Lian Duan, Yong Yao

**Affiliations:** ^1^ Key Laboratory of Endocrinology of National Health Commission, Department of Endocrinology, Peking Union Medical College Hospital, Chinese Academy of Medical Science and Peking Union Medical College, Beijing, China; ^2^ Department of Neurosurgery, Peking Union Medical College Hospital, Chinese Academy of Medical Science and Peking Union Medical College, Beijing, China

**Keywords:** thyrotropin-secreting adenoma, adolescent-onset, somatostatin analogs, transsphenoidal surgery, multidisciplinary therapy

## Abstract

**Background:**

Thyrotropin-secreting adenoma (TSH-oma) is a very rare kind of functional pituitary adenoma, especially that which occurs in adolescents. However, its potential clinical and therapeutic characteristics are still unknown.

**Objectives:**

The study was aimed to summarize the clinical and therapeutic characteristics of patients with adolescent-onset TSH-oma.

**Methods:**

We retrospectively analyzed six (4.1%) adolescent-onset TSH-oma cases from 148 patients who were diagnosed with TSH-oma at our hospital between January 2012 and October 2020. A literature review was performed on the PubMed online database, and 14 adolescent-onset TSH-oma cases were retrieved. Then, the characteristics of clinical manifestations, treatment outcomes, and follow-ups were analyzed and compared to the adult TSH-oma patients.

**Results:**

Altogether, 20 adolescent-onset cases were included in this study having mean onset age of 13.4 ± 3.3 years. Males were found to be slightly predominant (M: F = 1.5:1) in our study. The median baseline levels of TSH, FT3, and FT4 in adolescent-onset cases were found to be 6.30 [interquartile range (IQR) 9.82] µIU/ml, 9.18 (IQR 11.61) pg/ml, and 3.22 (IQR 1.90) ng/dl, respectively, which were all significantly higher than the adult patients of our hospital. Also, the adolescent-onset cases showed more large tumor ratio (36.8% vs. 9.3%, p = 0.007) compared to the adult patients. Compared to the patients of all ages in the literature, the biochemical remission rate of SSAs (57.1%) and remission rate of TSS (38.9%) were found to be considerably lower in adolescent-onset patients, while the recurrence rate (44.4%) was found to be considerably higher.

**Conclusions:**

Adolescent-onset TSH-oma patients showed higher TSH and thyroid hormone levels, more large tumors, and worse treatment outcomes than adult cases. Hence, early diagnosis, multidisciplinary therapy, and close follow-up should be highlighted to improve the prognosis.

## 1 Introduction

Thyrotropin (TSH)-secreting adenoma (TSH-oma) is a type of functional pituitary adenoma, which produces excessive amounts of TSH causing elevated levels of serum thyroid hormone. Also, it has a very rare occurrence and accounts for only 0.5 to 3% of all types of pituitary adenomas (1). The main manifestations of this disease are central hyperthyroidism and pituitary lesions. Most patients present with typical symptoms of hyperthyroidism, and inappropriate secretion of TSH which means elevated thyroid hormones with unsuppressed TSH levels. Further, pituitary macroadenomas usually result in visual field defects and vision loss. As Pit-1 lineage tumors, approximately 16% and 10% of the TSH-oma can co-secrete growth hormones (GH) and prolactin (PRL), respectively (2). Therefore, patients may also exhibit symptoms of gigantism, acromegaly, lactation, oligomenorrhea, etc. The first-line therapy for TSH-oma is transsphenoidal surgery (3). Further, somatostatin analogs can be used effectively to normalize the thyroid hormones, preoperatively (4). Adolescent-onset cases of TSH-oma are rare and have unknown potential differences between underage and adult TSH-oma patients. We presented the clinical features, treatments, and follow-ups of six adolescent-onset TSH-oma patients in our center along with reviewing 14 cases from the literature. Thus, we aimed to summarize the characteristics of adolescent-onset TSH-oma and gather some opinions about its management.

## 2 Methods

### 2.1 Diagnostic and Remission Criteria for TSH-Oma

The diagnosis of TSH-oma was established based on endocrinological and radiological evidence and was as follows: (1) presented with clinical symptoms of hyperthyroidism, (2) high levels of circulating total or free thyroid hormones in the presence of non-suppressed TSH levels and these evidences were repeatable, and (3) Enhanced Magnetic Resonance Imaging (MRI) identified a tumor in the pituitary region (3, 5). According to the maximum diameter of the tumor, the pituitary TSH-oma was classified into microadenomas (<10 mm), macroadenomas (≥10 mm), and large adenomas (≥30 mm).

The remission criteria included cured hyperthyroidism symptoms along with normalized TSH and thyroid hormone levels and resolution of neuroradiological lesions.

### 2.2 Collection of the Patients

A total of 148 patients were diagnosed with TSH-oma at our hospital between January 2012 and October 2020. Out of these, we retrospectively analyzed six (4.1%) adolescent-onset TSH-oma patients. This study was approved by the Ethics Committee of our hospital. The inclusion criteria were: (1) meeting the diagnosis criteria of TSH-omas; (2) symptom onset before the patient turned 18 years old; and (3) being operated in our center with available medical records. The exclusion criteria were: (1) having undergone total or subtotal thyroidectomy previously; and (2) suffering from other thyroid diseases, such as Grave’s disease.

We obtained the patients’ data from the electronic medical records system of our hospital. The information included: (1) the demographic features and clinical symptoms; (2) serological tests involving the measurement of the concentrations of the TSH, free tetraiodothyronine (FT4), free triiodothyronine (FT3), thyroxine (T4), triiodothyronine (T3), growth hormone (GH), insulin-like growth factor 1 (IGF-1), prolactin (PRL), adrenocorticotropic hormone (ACTH), cortisol (F), follicle-stimulating hormone (FSH), luteinizing hormone (LH), estradiol (E2), testosterone (T), serum calcium (Ca), serum phosphorus (P), alkaline phosphatase (ALP), 25-hydroxyvitamin D [25(OH)D], parathyroid hormone (PTH), fasting blood glucose (FBG), fasting insulin, total cholesterol (TC), triglyceride (TG), high-density lipoprotein cholesterol (HDL-C), and low-density lipoprotein cholesterol (LDL-C), which were tested using the standard methods at the department of clinical laboratory of our hospital (6); (3) imaging examinations which were as follows: enhanced 3.0T pituitary MRI, somatostatin receptor scintigraphy (^99^mTc labeled octreotide was administered as intravenous injection, then the whole-body scintigraphy was performed at 1 and 4 h after the administration), and thyroid ultrasound examination (Philips iU22, 8–15 MHz); (4) Octreotide suppression tests, which were performed using the 3-day method (Novartis octreotide was administered as subcutaneous injection with a dose of 0.1 mg q8h for 3 days. The TSH, FT3, and FT4 levels were measured at 0, 2, 4, 6, 8, 12, 24, 48, and 72 h. If the TSH levels decreased more than 50% during the tests, the patient was considered sensitive to octreotide); (5) pathological examinations included the following: H&E and immuno-histochemistry staining for pituitary hormones and transcription factors; and (6) treatments and outcomes. Finally, we enrolled six adolescent-onset and 107 adult-onset TSH-oma patients whose preoperative information was available at our center.

### 2.3 Literature Review

We conducted a literature search of the PubMed online database until January 1, 2021, and found 411 articles containing the following keywords: [thyrotropinoma] OR [TSH-secreting adenoma] OR [thyrotropin-secreting adenoma] OR [thyroid-stimulating hormone-secreting adenoma]. The age filters were set as [child: birth-18 years] or [young adults: 19–24 years]. The exclusion criteria were as follows: (1) the age of the patient being older than 18 years at the symptom onset; (2) cases not being reported in English; and (3) no new cases being presented in the literature.

### 2.4 Statistical Analyses

Clinical data were analyzed using IBM SPSS v.23 (IBM Corporation, NY, USA). Categorical data were compared using a χ^2^ or Fisher’s exact test. The normality of continuous variables was analyzed by the Shapiro–Wilk test. Normally or non-normally distributed variables were compared using a student t-test or non-parametric Mann–Whitney U test. Data were presented as mean ± SD, or median (IQR). P <0.05 was considered statistically significant.

## 3 Results

### 3.1 The Presentation of Six Cases of TSH-Oma

#### 3.1.1 Demographics and Baseline Characteristics

**Figure 1:**
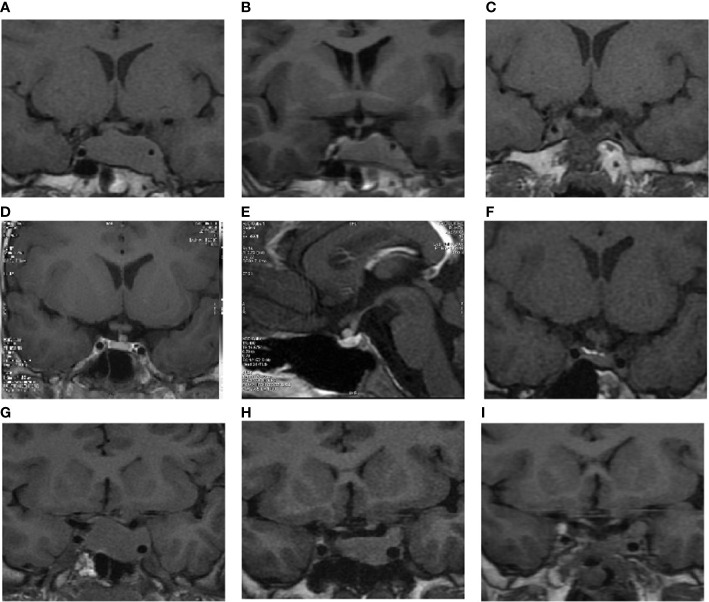
The pituitary MRI imaging of cases 1, 2, and 3. **(A)** Coronal imaging of case 1 during the first visit. **(B)** Coronal imaging of case 1 after long-term SSA therapy. **(C)** Coronal imaging of case 1 during the last follow-up. **(D)** An ectopic pituitary stalk tumor in case 2 during the first visit. **(E)** Sagittal pituitary imaging of case 2 during the first visit. **(F)** Coronal pituitary imaging of case 2 during the last follow-up. **(G)** Coronal imaging of case 3 during the first visit. **(H)** Coronal imaging of case 3 after long-term SSA therapy. **(I)** Coronal imaging of case 3 at the last follow-up.

In our study, we enrolled six adolescent-onset patients from our hospital (**Table 1**). The corresponding ages of the patients ranged between 6.8 and 17.0 years at the symptom onset, while the female/male ratio was 2:1. The delay in diagnosis was 76.7 ± 67.3 months on an average (4–180 m). Except for case 3 who was diagnosed as TSH-GH-PRL mixed adenoma, all others were pure TSH-secreting adenomas with hyperthyroid symptoms such as polyphagia, sweating, hand tremor, and goiter. On pituitary MRI, four cases showed macroadenomas, out of which two were invasive (Knosp grade 4). The other two showed microadenomas, with one of them being ectopic and located on the left front of the pituitary stalk (**Figure 1**) (7). No patients were presented with headaches or visual impairments.

**Table 1:** Clinical features, examinations, treatments, and outcomes of six adolescent-onset TSH-oma cases at our center.

	Case 1	Case 2	Case 3	Case 4	Case 5	Case 6
Sex	F	F	M	F	M	F
Age at Onset (years)	6.8	8.3	15.0	15.0	17.0	17.0
Age at Diagnosis (years)	7.1	10.3	18.0	23.0	32.0	27.0
Ht (cm)	127.0	152.0	182.0	164.0	175.0	165.0
Wt (kg)	24.0	46.0	86.0	59.0	80.0	65.0
BMI (kg/m^2^)	14.9	19.9	26.0	22.0	26.1	23.9
Diagnosis Delay (months)	4	24	36	96	180	120
Symptoms at Onset	Polyphagia, Heat intolerance	Polyphagia, Goiter	Abnormal growth of face, hands, and feet	Sweating, Palpitation	Sweating	Hand tremor, Heat intolerance
Hypopituitarism	No	No	Hypogonadism	No	No	No
**Laboratory examination at the diagnosis of TSH-omas**						
TSH (µIU/ml)	9.949	2.772	3.423	3.47	12.251	4.815
FT3 (pg/ml)	>20	6.43	9.47	5.28	11.57	7.37
FT4 (ng/dl)	6.249	2.12	3.263	2.04	3.18	2.7
T3 (ng/ml)	>8	2.15	3.429	1.64	3.26	ND
T4 (µg/dl)	>30	11.9	26.6	13.2	13.33	ND
GH (ng/ml)	1.7	0.2	59.1	0.7	<0.5	1.4
IGF-1 (ng/ml)	104	388	773	498	165	456
IGF-1 SDS	−1.58	0.75	3.73	2.92	−0.48	3.67
PRL (ng/ml)	5.27	ND	145.4	16.37	7.2	12.77
TSH suppression rates in OST	70.00%	73.00%	44.80%	88.90%	27.50%	90.50%
**Imaging, IHC staining, and other examinations**						
Pituitary Tumor Size (mm)	12 × 31 × 13	4.4	23 × 18 × 21	3.5 × 2	11 × 8.6	15 × 17 × 15
Knosp grade	4	0	4	1	2	2
SSTR scintigraphy	Positive	Negative	Positive	Negative	Positive	ND
Thyroid Ultrasound	Increased thyroid blood flow, consistent with hyperthyroidism	multiple cystic nodules with crystals, uneven echo	Thyroid enlargement, diffuse lesions, solid-cystic nodules in right lobe	Uneven echo, diffuse patchy hypoechoic areas	Diffuse lesions, multiple hypoechoic areas	Uneven echo, multiple cyclic nodules
Thyroid Palpation	II° enlargement	III° enlargement	0–I° enlargement	II° enlargement	I° enlargement	II° enlargement
IHC staining	TSH+/GH+/PRL−	TSH+/GH+/PRL−	TSH-/GH+/PRL−	TSH+/GH+ /PRL+	TSH+/GH+ /PRL−	ND
Ki-67 index	3%	<1%	1%	<1%	<1%	ND
**Treatments and outcomes**						
Preoperative medical therapy	Octreotide, Thyrozol	Octreotide	Octreotide, Bromocriptine	Octreotide	Octreotide	Octreotide
Surgery	Partly TSS	TSS	Partly TSS	TSS	TSS	TSS
Surgery Outcomes	Euthyroid, Relapsed 2 m later	Remission, Intracranial infection, CSF linkage.	No biochemical remission	Remission	Remission	Remission
Subsequent therapy	γknife + thyrozol→ second TSS→ sellar regional RT	Rhinosphenoid sinus repair surgery	RT	Propranolol	No	No
Outcomes	No biochemical remission	Remission	Euthyroid, Elevated GH and PRL levels	Remission	Remission	Remission
Follow-up time (year)	4	2	0.5	1	4	2

OST, Octreotide suppression tests; SSTR scintigraphy, somatostatin receptor scintigraphy; TSS, transsphenoidal surgery; CSF, cerebrospinal fluid; RT, radiotherapy; ND, not detected.

Normal ranges: TSH: 0.38–4.34 µIU/ml, FT4: 0.81–1.89 ng/dl, FT3: 1.80–4.10 pg/ml, T3: 0.66–1.92 ng/ml, T4: 4.30–12.50 µg/dl, GH: <2.0 ng/ml, PRL<30 ng/ml.

During their first visits, all patients were presented with elevated levels of serum FT3, FT4, and non-suppressed TSH levels (**Table 1**). The average value of the TSH, FT3, and FT4 were 6.11 ± 3.99 µIU/ml, 10.02 ± 5.38pg/ml, and 2.94 (1.90) ng/dl, respectively.

In terms of the development, two out of six (33.3%) patients were diagnosed before 10 years of age and had a height of +0.9 SD/+1.89 SD and weight of +0.34 SD/+2.31 SD, which was comparable to the same age and sex. The BMI of the other four cases at diagnosis was 24.5 ± 2.0 kg/m^2^ on average. Especially, case 3 was overweight, showed hypogonadism, and had high fasting insulin which could be attributed to the patient’s elevated levels of serum GH, IGF-1, and PRL. As for complications, pure TSH-oma cases showed normal glucose, lipids, and bone turnover indexes. Three out of the six (50.0%) cases suffered from arrhythmia, while two out of the six (33.3%) cases had mild valvular disease (**Table 1**).

Four out of the six (66.7%) cases were sensitive to octreotide sensitive suppression tests, with TSH suppression rates ranging between 70.0 and 90.5%. The TSH suppression rates in the two insensitive cases were 44.8 and 27.5%. However, in case 5, whose TSH level decreased 27.5%, the octreotide suppression test was discontinued at the eighth hour due to the high cost of Sandostatin. In the five cases that received somatostatin receptor scintigraphy, three (60.0%) cases showed radioactive concentration in the pituitary region while others showed no radioactive concentration in the pituitary region.

#### 3.1.2 Medical Therapy

To normalize thyroid functions before the operation, all patients underwent medical therapies with somatostatin analogs (SSAs) before surgery. Due to the SSAs treatment, three cases (50.0%) became euthyroid. Another patient also turned euthyroid after using the combination treatment of SSA and bromocriptine. However, even after the SSAs therapy, the thyroid hormones in the two other cases (cases 1 and 6) showed only a decrease and were still abnormal. We used combined thyrozol treatment in the following month for case 1, while interrupted medical therapy in case 6 for a skin rash and endermic induration caused by SSA.

#### 3.1.3 Surgery Therapy and Immunohistochemical (IHC) Staining

Endoscopic transsphenoidal pituitary tumor resection and sellar reconstruction (TSS) were performed at our hospital by an experienced neurosurgery team. The cerebrospinal linkage happened in two cases (case 2: postoperative, secondary to intracranial infection; case 3: intraoperative). Also, two out of six (33.3%) tumors were tough, while four out of six tumors (66.7%) had soft consistencies. The H&E staining was performed in all cases, while IHC staining was performed in five cases (cases 1–5). The H&E staining confirmed that the tumors were adenomas. TSH staining was positive in all the cases except in case 3, which might be due to the inappropriate sampling during the process of pathological sectioning. Only case 4 was stained by Pit-1, which showed a positive result. The Ki-67 index in case 1 was approximately 3%, while it was less than or equals to 1% in all other cases. P53 staining was negative in all cases.

#### 3.1.4 Postoperative Therapy and Follow-Up

After performing the TSS, four cases were in remission during their 1 to 4 years of follow-up (**Table 1**). Case 1, whose macroadenoma was partly removed, relapsed two months after the TSS. Hence, γ-knife radiosurgery was performed, and antithyroid therapy was provided for two years, but the patient’s residual tumor enlarged. Then, a second TSS was performed, resulting in temporary euthyroidism and relapse after two months. Next, regular sellar radiotherapy was performed. In case 3, the thyroid hormones were gradually reduced to normal levels after the TSS, but the GH, IGF-1, and PRL concentrations remained elevated. Thus, sellar radiotherapy was performed.

### 3.2 Literature Review

We found 14 adolescent-onset TSH-oma patients through elaborate retrieval from literature reported between 1964 and 2016 (**Table 2**). The average disease onset age was 13.5 ± 2.8 years, and the gender ratio was 2:5 (F/M). Of the 14 patients, 11 (18.6%) cases complained about hyperthyroid symptoms such as weight loss, hand tremors, and tachycardia, while six out of the 14 (42.9%) cases were presented with headaches, hemianopsia, or vision changes. Hypopituitarism was exhibited in one case. The mean diagnosis delay was 8.0 (28.0) months (0.75–72 m). Of the 14 patients, 12 (85.7%) cases had macroadenomas, of which six were large adenomas. Additionally, two cases were diagnosed as GH-TSH and GH-PRL-TSH secreting pituitary adenomas. During their first visits, the average concentrations of TSH, FT3, and FT4 were found to be 10.51 ± 7.45 µIU/ml, 9.18 (81.91) pg/ml, and 3.40 (2.67) ng/dl, respectively. No significant differences were observed in the baseline hormone levels, and tumor sizes between adolescent-onset cases enrolled in our center and cases reviewed (**Table 3**).

**Table 2:** Basic characteristics and treatment outcomes of 14 retrieved cases.

Cases	Onset Age/Sex	Symptoms	Diagnosis delay (m)	TSH/FT3/FT4/T3/T4	Tumor size (mm)	Hypopituitarism	Treatments	Pre-op MEDs	First Surgery Outcomes	Ki-67 index	Outcomes	Follow-up time (m)
7 (8)	8/M	Emaciation, Muscle weakness	2	13.17/113/3.8/NA/NA	Macro	No	TSS→LT4	No	Remission	NA	Remission	11
8 (9)	11/F	Headache, hemianopsia	24–36	NA	Large	NA	PTU→Craniotomy→death	III	NA	NA	Death	NA
9 (10)	11/F	Headache, Polyuria, Polydipsia, Diplopia, Hemianopia	8	NA	Large (48 × 62 × 58)	NA	craniotomy→RT→carbimazole	No	No remission	NA	No remission	20
10 (11)	11/M	Autoimmune polyglandular syndrome, Tachycardia	NA	16.8/18.12/3.52/NA/NA	Large (26 × 32)	No	TSS→RT→GH/LT4	No	Remission for 3 months	NA	Remission	72
11 (12)	12/F	Goiter, Sinus tachycardia, Tremors	18	21.11/9.25/2.21/NA/NA	Micro (6 × 5)	No	Thyrozol→TSS	III	Remission	NA	Remission	14
12 (13)	13/F	Poor weight gain, Pubertal delay	72	3.0/NA/NA/5.29/15.93	Micro (9)	NA	PTU→thyroidectomy→LT4→TSS→LT4	II	Remission	NA	Remission	24
13 (14)	13/M	School performance deterioration, Secondary enuresis	Several	9.8/NA/7.7/6/>24	Macro (20 × 15)	GH/ADH	PTU+SSA→TSS	III	NA	NA	Death	NA
14 (15)	13/M	Increased height velocity, Weight loss, Polyphagia, Visual impairment	6	3.54/6.75/1.94/2.48/NA	Large (40 × 45)	NA	MMI→TSS*3→SSA→SSA+RT	NA	Remission for 2 months	11%	Hypothyroidism	7
15 (16)	15/M	Weight loss, Irritability, Difficulty sleeping, Headache	0.75	24.8/154.08/31/27.25/54.2	Large (30 × 30 × 30)	NA	PTU→TSS→RT	III	Remission for 1.5 months	NA	No remission	1.5
16 (17)	15/M	Headache, Weight loss	36	6.49/NA/NA/3.16/24.6	NA	No	craniotomy→PTU→SSA (in the plan)	No	No remission	NA	No remission	3
17 (18)	16/M	Mild thyrotoxicosis, Intermittent dizziness	8	1.8/8.31/3.28/NA/NA	Macro	No	TSS→BCT + Pergolide→SSA	No	Remission for 1 months	NA	No remission	5
18 (19)	16/M	Goiter, Hypertension	NA	13.6/NA/4.35/NA/NA	Macro (17 × 15)	No	MMI→TSS→MMI→SSA	III	Remission for 1 months	5–10%	Remission	48
19 (20)	17/M	Paroxysmal headache, Blurred vision, Palpitation, Hand tremor, Sweating	4	5.934/7.06/2.97/NA/NA	Large (44 × 39)	NA	SSA→TSS→γ knife RT	I	Remission for 4 months	<1%	Remission	24
20 (21)	18/M	NA	NA	6.1/9.11/2.62/NA/NA	Macro (22)	NA	TSS→SSA→TSS	NA	Remission for 5 years	NA	Remission	84

TSH, µIU/ml; FT3, pg/ml; FT4, ng/dl; T3, ng/ml, T4, µg/dl.

The outcomes of preoperatively medical therapy (pre-op MEDs) were classified into 3 grades: I: TSH and thyroid hormones normalized, no symptom or tumor size worsened; II: TSH and thyroid hormones decreased but still abnormal, no symptom or tumor size worsened; III: TSH or thyroid hormones levels rose, symptoms worsen, or tumor enlarged.

Na, not available; TSS, transsphenoidal surgery; LT4, levothyroxine; SSA, somatostatin analogs; PTU, propylthiouracil; MMI, methimazole; RT, radiotherapy; BCT, bromocriptine; ADH, antidiuretic hormone.

**Table 3:** The comparison between adolescent-onset cases at our center and reviewed cases from literature along with the comparison between adolescent-onset cases and adult cases.

	Adolescent cases in our center (n = 6)	Adolescent cases reviewed (n = 14)	P-values	Adolescent-onset cases (n = 20)	Adult cases (n = 107)	P-values
Age	13.2 ± 4.5	13.5 ± 2.8	0.848	13.4 ± 3.3	38.8 ± 11.7	0.000**
Diagnosis delay	76.7 ± 67.3	8.0 (28.0)	0.050	21.0 (58.5)	24.0 (66.0)	0.570
TSH	6.11 ± 3.99	10.51 ± 7.45	0.199	6.30 (9.82)	4.39 (3.19)	0.012*
FT3	10.02 ± 5.38	9.18 (81.91)	0.439	9.18 (11.61)	5.84 (3.51)	0.000**
FT4	2.94 (1.90)	3.40 (2.67)	0.329	3.22 (1.90)	2.25 (1.13)	0.007**
T3	3.70 ± 2.52	5.29 (13.81)	0.347	3.34 (4.10)	2.01 (1.08)	0.001**
T4	19.01 ± 8.59	29.68 ± 16.82	0.253	23.74 ± 13.21	13.91 (5.27)	0.026*
Tumor max diameters (mm)	14.98 ± 10.81	28.70 ± 17.58	0.109	23.56 ± 16.48	18.07 ± 10.40	0.216

TSH, µIU/ml; FT3, pg/ml; FT4, ng/dl; T3, ng/ml; T4, µg/dl.

*p < 0.05, **p < 0.01.

In the treatment aspect, eight out of the 14 (57.1%) patients received preoperative medication therapies. However, except for one patient who was treated with lanreotide (40 mg, q2w), all other antithyroid preoperative preparations failed because of much higher TSH levels, emerging tumor compression symptoms, tumor enlargement or goiter progression. All patients received surgery therapy, but only three patients attained long-term remission. Of the 14 patients, two died postoperatively, while nine showed no remission or relapses. Then they received repeated surgeries, SSAs, RTs, and (or) antithyroid therapies. Four patients were still hyperthyroid during their last follow-up, including one who attained remission but relapsed after the SSA interruption.

### 3.3 Comparison Between Adolescent-Onset and Adult Cases

Compared to the 107 adult TSH-oma patients admitted to our hospital, the adolescent cases (n = 20) showed significantly higher baseline levels of TSH, FT3, FT4, T3, and T4 than that of the adult patients (p = 0.012, p = 0.000, p = 0.007, p = 0.001, and p = 0.026), whose values were 6.30 (9.82) µIU/ml, 9.18 (11.61) pg/ml, 3.22 (1.90) ng/dl, 3.34 (4.10) ng/ml, and 23.74 ± 13.21 µg/dl, respectively. Also, the large tumor ratio was higher compared to that of the adult cases (36.8% vs. 9.3%, p = 0.007) (**Table 3**). No significant differences were observed in the gender ratio, diagnosis delay, and heart complication incidences between adolescent-onset and adult patients in our hospital (gender: p = 0.144, diagnosis delay: p = 0.570, arrhythmia incidence: p = 0.486, valvular disease incidence: p = 0.350) (**Table 4**).

**Table 4:** The comparison of gender ratio, baseline tumor types, and complications between adolescent-onset and adult cases.

		Adolescent-onset cases		Adult cases in our center		χ2	P
		number	%	Number	%		
Sex	M F	12 8	60.0% 40.0%	44 63	41.1% 58.9%	2.436	0.144
Tumor type	Large	7	36.8%	10	9.3%	9.086	0.007**
	Macro	8	42.1%	74	69.2%		
	Micro	4	21.1%	23	21.5%		
Arrhythmia	Yes	3	50.0%	26	40.6%	/	0.486
	No	3	50.0%	38	59.4%		
Value diseases	Yes	2	33.3%	35	50.7%	/	0.350
	No	4	66.7%	34	49.3%		

**p < 0.01.

## 4 Discussion

TSH-oma is extremely rare, especially in adolescent cases. Herein, we described six cases in our center and 14 cases retrieved from literature to summarize the characteristics of clinical manifestations and therapeutic outcomes in adolescent-onset TSH-oma patients. Of the 20 cases, 16 (80.0%) showed macroadenomas and four (20.0%) showed microadenomas, which was consistent with the overall 70–90% macroadenomas ratio summarized before (1). However, adolescent-onset patients may have more large pituitary tumors than adult patients (36.8% vs 9.3%) along with significantly higher baseline TSH, FT3, FT4, T3, and T4 levels (**Table 3**). Both of these observations contribute to the therapeutic challenges seen in adolescent-onset patients. In the case of complications, five pure TSH-oma cases in our center showed normal height growth and puberty development and also the lipid level and bone turnover indexes. Arrhythmia and valvular diseases were seen in more than 30% of the TSH-oma cases at our center. Recently, a meta-analysis revealed that atrial fibrillation or heart failure happened in 11.1% of 535 adult TSH-oma cases (22). Another research found that TSH-oma can significantly induce left atrial enlargement and subclinical atrial fibrillation since excess thyroid hormones can increase the arrhythmogenic activity of the pulmonary veins, and increase the hemodynamic load (23). Thus, the potential cardiovascular complications caused by the TSH-oma may be more common than reported and should be taken seriously.

SSAs can normalize thyroid functions without increasing the TSH levels, and reduce the operative difficulties. Thus, it is generally applied when the tumor is large or invasive. Compared to the 90% (24) or 73–100% (1) remission rates reported in all age patients, the effectiveness of SSAs can be considered worse in adolescent patients considering that only four out of seven (57.1%) patients achieved biochemical remission. For intractable cases, combination medical therapy such as SSAs combined with dopamine analogs and (or) antithyroid drugs may work well. There is a need to be cautious while using antithyroid drugs alone preoperatively because it may increase TSH levels through feedback regulation (19).

SSAs bind with high affinity to SSTR2 and lower affinity to SSTR3 and SSTR5, making *in vivo* effects by activating these specific SSTR subtypes (25). It is suggested that SSAs may inhibit TSH secretion in all TSH-omas that express SSTR2 while the coexistence of SSTR5 can enhance the effectiveness of SSAs (26, 27). Thus, the difference in the expression levels of SSTR5 and SSTR2 in TSH-omas may explain or even predict the different outcomes of treatment with SSAs. However, since SSTR staining has not yet been deployed in our center, the data on its expression remain unavailable in our cases.

TSS is the first-line therapy for TSH-omas. In the 20 cases, seven out of 18 (38.9%) were in remission, three out of 18 (16.7%) didn’t relieve, while eight out of 18 (44.4%) relapsed. Two cases died from postoperative infection, probably due to failed primary therapies and limited surgical techniques used. The recurrences in adolescent-onset patients are more frequent than the overall 0–21.4% recurrence rates observed (28–31). Similarly, the cavernous sinus invasion and larger tumor size are related to the tendency of recurrence (28). Only three out of 14 (21.4%) macroadenoma patients attained remission after one TSS, while the remission rate in microadenoma patients was 100%. For relapsed cases, repeat surgeries, radiotherapies, and (or) SSAs were applied. Overall, relatively high occurrence rates highlight the importance of having multidisciplinary therapy and close follow-up in adolescent patients.

The results of IHC showed that a special case diagnosed as TSH-GH-PRL mixed adenoma was found to be negative for TSH staining. However, the normalization of his thyroid functions after TSS suggested correct clinical diagnosis, so the negative pathological result may be due to inappropriate tissue sampling. Also, the positive staining of GH and (or) PRL in pure TSH-oma patients was actually understandable because positive IHC staining did not necessarily mean the hypersecretion of hormones, such as silent pituitary adenoma (4). Ki-67 index of case 1 was approximately 3% with the tumor invading into the cavernous sinus and surrounding the left internal carotid. According to recent studies, Ki-67 is a cell proliferation-associated antigen. A higher Ki-67 index usually means more aggressive tumor behavior and more recurrence risk with 2.5–3% cut-off points for pituitary adenomas (32–35). The treatment for TSH-oma in the case of higher Ki-67 index was also more challenging, for example, in one case where the Ki-67 index was 11%, three TSSs had to be performed along with providing SSAs and RTs. However, whether the Ki-67 index is generally higher in adolescent patients or not is still unknown. Meanwhile, it is necessary to understand the genetic background of TSH-oma tumorigenesis in early-onset patients. Current research has found a very uncommon germ-line MEN1 and AIP mutation in familial cases (4) and some somatic mutations and copy number changes in sporadic cases (36). But no oncogenes or proto-oncogene mutations have been identified (4). However, these findings are very limited in clarifying the molecular mechanisms of TSH-oma and need further studies. Additionally, our study has some limitations. As a retrospective study, some data are missing causing a loss of information about the follow-up. The postoperative heart functions, thyroid ultrasonographic manifestations, and glucolipid metabolisms have not been followed up adequately. Also, some studies were published long ago; hence, the diagnosis and treatments may be non-standard. Moreover, the indiscoverable pituitary microadenomas may cause misdiagnosis of TSH-oma along with a statistical bias.

### Conclusion

In conclusion, adolescent-onset TSH-oma patients have higher baseline TSH and thyroid hormone levels, more large tumors, lower biochemical remission rates of SSAs, lower surgery remission rates, and higher postoperative recurrence rates than seen in the adult cases. Whether in primary therapies or postoperative management, more difficulties are faced in treating adolescent-onset patients, especially those with macroadenomas. Hence, early identification, preoperative SSA application, multidisciplinary therapy, and close follow-up can improve patients’ prognoses and should be highlighted.

## Data Availability Statement

The raw data supporting the conclusions of this article will be made available by the authors, without undue reservation.

## Ethics Statement

The studies involving human participants were reviewed and approved by the Ethics Committee of Peking Union Medical College Hospital. Written informed consent from the participants’ legal guardian/next of kin was not required to participate in this study in accordance with the national legislation and the institutional requirements.

## Author Contributions

YMY wrote the manuscript and prepared figures and tables. YMY and JL collected the data. KD, LL, HJZ, XLL, XJB, LD, and YY diagnosed and provided treatments for the cases involved in this study. HJZ, LD, and YY designed the study and provided suggestions for the manuscript writing. All authors contributed to the article and approved the submitted version.

## Funding

This work was supported by the Chinese Academy of Medical Sciences Innovation Fund for Medical Science (CAMS-2016- I2M-1-002).

## Conflict of Interest

The authors declare that the research was conducted in the absence of any commercial or financial relationships that could be construed as a potential conflict of interest.

## Publisher’s Note

All claims expressed in this article are solely those of the authors and do not necessarily represent those of their affiliated organizations, or those of the publisher, the editors and the reviewers. Any product that may be evaluated in this article, or claim that may be made by its manufacturer, is not guaranteed or endorsed by the publisher.
